# Perioperative Immunonutrition in Well-Nourished Patients Undergoing Surgery for Head and Neck Cancer: Evaluation of Inflammatory and Immunologic Outcomes

**DOI:** 10.3390/nu5041186

**Published:** 2013-04-09

**Authors:** Amy Turnock, Philip C. Calder, Annette L. West, Mark Izzard, Randall P. Morton, Lindsay D. Plank

**Affiliations:** 1 Department of Surgery, University of Auckland, Auckland 1142, New Zealand; E-Mail: amymadeline@gmail.com; 2 Human Development and Health Academic Unit, Faculty of Medicine, University of Southampton, Southampton SO16 6YD, UK; E-Mails: pcc@soton.ac.uk (P.C.C.); a.west@soton.ac.uk (A.L.W.); 3 Department of Otorhinolaryngology, Auckland City Hospital, Auckland 1142, New Zealand; E-Mail: MarkI@adhb.govt.nz; 4 Department of Otolaryngology, Counties-Manukau District Health Board, Auckland 1640, New Zealand; E-Mail: RPMorton@middlemore.co.nz

**Keywords:** immunonutrition, arginine, omega-3 fatty acids, fish oil, surgery, head and neck cancer, inflammation, cytokines

## Abstract

Limited work is available on the benefits of nutritional support enriched with arginine and *n*-3 fatty acids in surgical patients with head and neck cancer, particularly if well-nourished. We conducted a pilot study in these patients to examine effects on inflammatory markers and clinical outcome. Patients scheduled for radical resection of the oral cavity were randomised to 5 day preoperative and 5 day postoperative Impact^®^ (IMN, *n* = 4), or no preoperative supplementary nutrition and Isosource^®^ postoperatively (STD, *n* = 4). Plasma fatty acids, C-reactive protein (CRP), tumour necrosis factor (TNF)-α, interleukin (IL)-6 and IL-10 were measured at baseline, day of surgery and on postoperative days (POD) 2, 4 and 10. Postoperative complications were recorded. The (eicosapentaenoic acid plus docosahexaenoic acid) to arachidonic acid ratio was significantly higher in IMN patients on POD 2, 4 and 10 (*P* < 0.01). While not statistically significant, CRP, TNF-α, and IL-6 concentrations were higher in the STD group on POD2 while IL-10 was lower. Median length of stay was 10 (range 10–43) days in the IMN group and 21.5 (7–24) days in the STD group. Five complications were seen in the STD group and two in the IMN group. The results support the need for a larger trial focusing on clinical outcome.

## 1. Introduction

Immunonutrition based on *n*-3 fatty acids from fish oil with arginine and/or glutamine provided as a pre-, peri-, or postoperative supplement has been shown in meta-analysis to significantly reduce postoperative infectious complications and length of hospital stay in patients undergoing, predominantly, gastrointestinal surgery [1,2,3,4]. Malnourished gastrointestinal surgery patients appear to benefit from this therapy whether provided pre- or perioperatively [5] or postoperatively only [6]. The expectation that normally nourished patients may also benefit, possibly because of the down-regulation of the inflammatory responses to surgery and amelioration of the postoperative immune depression associated with this treatment [7], has not been convincingly demonstrated. Gianotti *et al.* [8] showed both pre- and perioperative application of an immunonutritional supplement improved clinical outcome in well-nourished gastrointestinal surgery patients while no significant benefits were seen in more recent studies when the treatment was provided postoperatively only [9,10] or preoperatively only [11]. 

Application of these immunonutrition formulas in head and neck cancer patients undergoing surgery has been limited to date. Perioperative administration of a formula providing *n*-3 fatty acids and arginine reduced postoperative infectious complications but not length of hospital stay in a randomized trial reported by Snyderman *et al.* [12]. Casas-Rodera *et al.* [13] showed that this formula, when provided postoperatively only, did not confer statistically significant benefits for clinical outcome. In both of these studies, patients were not selected on the basis of nutritional status and were likely to be predominantly malnourished given the high prevalence of malnutrition in patients with head and neck cancer presenting for surgery [14]. Perioperative treatment with the same formula in a randomised trial reported by Felekis *et al.* [15] was associated with a reduction in complications both in the study group as a whole and in a well-nourished subgroup (weight loss <10% during previous 6 months).

The aim of the current study was to examine the effects of perioperative treatment with a formula providing *n*-3 fatty acids and arginine on fatty acid status, inflammatory markers, immune status and clinical outcome in non-malnourished patients scheduled for radical resection of the oral cavity, pharynx or larynx.

## 2. Materials and Methods

### 2.1. Patients and Study Protocol

The study was a non-blinded prospective randomized controlled trial carried out between May 2007 and January 2008 in patients who were scheduled for radical resection of the oral cavity, pharynx or larynx and who were expected to require artificial feeding by the enteral route postoperatively. Patients were excluded if they were: aged <16 year, malnourished (weight loss ≥10% of body weight within the last 6 months), had undergone previous wide-field radical radiotherapy, or were pregnant, diabetic or immuno-suppressed. The study was approved by the Auckland Ethics Committee (NTY/0610/094). The trial was registered with the Australian & NZ Clinical Trials Registry (ACTRN12607000162415).

Patients were recruited from the head and neck outpatient clinics at Auckland City Hospital and allocated sequentially by means of opaque sealed envelopes to immunonutrition (IMN) or standard treatment (STD) groups in a 1:1 ratio. The allocation sequence was derived from a computer-generated random enumeration. A baseline assessment approximately one week prior to hospital admission involved a visit to the Body Composition Laboratory in the Department of Surgery for measurement of total body protein (TBP) as an objective measure of malnutrition. At this time blood samples were obtained for determination of plasma fatty acids and markers of inflammation and immune status. These determinations were repeated for blood samples taken on day of surgery, immediately preceding induction of anaesthesia, and on postoperative days (POD) 2, 4 and 10. Whole blood samples were sent to the hospital laboratory for C-reactive protein (CRP), immunoglobulin and full blood count determinations. Plasma was immediately separated from cells by centrifugation and frozen at −80 °C until analysis. All in-hospital postoperative complications and dates of discharge were recorded by the same investigator (AT).

### 2.2. Nutrition

IMN patients were provided with three 74 g sachets per day of powdered Oral Impact^®^ (Novartis Consumer Health, Nyon, Switzerland) to be taken for 5 days immediately preceding day of surgery which when reconstituted with water yields 900 mL (1 kcal/mL) containing 11.3 g arginine, 6.1 g *n*-3 fatty acids (3.0 g as eicosapentaenoic acid (EPA) and docosahexaenoic acid (DHA)) and 1.2 g ribonucleic acid. Energy distribution of this formula is 22% protein, 53% carbohydrate and 25% fat. STD patients were not provided with any preoperative nutritional supplement. In both groups, postoperative feeding began as soon as tolerated via an intra-operatively placed nasogastric tube. IMN patients received enteral Impact^®^ (Novartis) and STD patients received our standard hospital enteral nutrition (Isosource Standard^®^, Novartis). The infusion rates were progressively increased up to full caloric requirements determined by the hospital dietitian as 25–30 kcal/kg. Where tube feeding was discontinued in the IMN group, nutritional support was continued with Oral Impact until at least POD5. Postoperative fluid intakes were recorded by ward staff. Composition of the oral and enteral feeds is given in Table 1.

**Table 1:** Composition of oral and enteral diets.

	Oral Impact	Enteral Impact	Isosource Standard
Energy (kcal/L)	1000	1000	1200
Protein (g/L)	56	56	43
Carbohydrate (g/L)	134	130	170
Fat (g/L)	28	28	39
Arginine (g/L)	12.6	12.5	0
Nucleotides (g/L)	1.3	1.2	0
EPA + DHA (g/L)	3.3	1.7	0
Fibre (g)	10	0	0

### 2.3. Total Body Protein

TBP was determined by prompt gamma *in vivo* neutron activation analysis as described in detail elsewhere [16]. For each patient, a pre-illness TBP was estimated based on height, sex, age and pre-illness body weight using equations developed in our laboratory from measurements on 386 healthy volunteers (163 M, 223 F, age range 17–82 years) [17]. Pre-illness weight was that recalled by the patient. The ratio of measured to pre-illness TBP was used as a measure of malnutrition. For the healthy controls, this ratio was 1.00 with the 2 SD limits, being 0.82–1.18. A ratio above 0.82 in a patient is indicative of normal protein status. 

### 2.4. Biochemistry

#### 2.4.1. Fatty Acids

Plasma phosphatidylcholine (PC) fatty acid composition was assessed by gas chromatography as described in detail elsewhere [18]. In brief, total lipids were extracted from 500 μL plasma using chloroform:methanol (2:1, v/v) containing 50 mg/L butylated hydroxytoluene, and 1 M NaCl followed by centrifugation at 800 *g* for 10 min. The lower lipid phase was collected and dried under nitrogen. Isolation of the PC fraction was performed using solid phase extraction. Total lipid was dissolved in dry chloroform and applied to a Bond Elut-NH2 cartridge (Varian Ltd, Oxford, UK). The column was washed with dry chloroform and the PC fraction was eluted using chloroform:methanol (60:40, v/v). Samples were dried and redissolved in toluene. Fatty acid methyl esters were produced by adding methanol containing 2% (v/v) H_2_SO_4_ and heating at 50 °C overnight. Samples were neutralised with 0.25 M KHCO_3_, 0.5 M K_2_CO_3_. The PC-fatty acid methyl esters were extracted by adding toluene, vortexing and centrifuging at 200× *g* for 2 min. They were then collected from the upper phase and dried under nitrogen. Samples were redissolved in hexane and fatty acid methyl esters resolved using a Hewlett Packard 6890 Gas Chromatograph (Agilent, Cheshire, UK) equipped with a 30 m × 0.25 μm × 0.25 mm BPX-70 fused silica capillary column. Fatty acid methyl esters were identified by comparison of retention times with those of authentic standards. The concentrations of each fatty acid were determined by the area under the peak using ChemStation software (Agilent) and each fatty acid is expressed as a percentage of the total. 

#### 2.4.2. Inflammatory Markers

Simultaneous quantification of TNF-α, IL-6 and IL-10 was undertaken using a high sensitivity human cytokine multiplex immunoassay kit (LINCO Research, Inc., St. Charles, MO) and the Luminex micro-beads array system using the manufacturer’s instructions (Luminex Corp., Austin, TX). The antibody specific to each cytokine is covalently coupled to a different Luminex micro-bead uniquely labeled with a fluorescent dye. The micro-beads were incubated with standards, controls and samples in a 96-well microtiter plate on a plate-shaker overnight at 4 °C. After incubation the plates were washed and a detection cocktail was added to each well as a mixture containing each of the antibodies. After 1 h incubation with agitation at room temperature, streptavidin-phycoerythrin was added to each well and incubated with agitation for an additional 30 min at room temperature. After a final wash step, the beads were resuspended in buffer and read on the Luminex instrument. All samples were tested in duplicate wells and the means of the duplicates reported. When the concentrations were less than the detection limit of the assay the values of the limit were included in the data analysis. All plasma samples were clarified by centrifugation at 14,000 rpm for 10 min at 4 °C in a refrigerated microfuge immediately prior to analysis. High sensitivity CRP was measured by the hospital laboratory using laser nephelometry.

#### 2.4.3. Immune Status

Immunoglobulin A, G and M concentrations in serum and total number of lymphocytes were measured by the hospital laboratory using standard techniques.

### 2.5. Clinical Outcome

Assessment of clinical outcome was undertaken until discharge and included postoperative complications and length of hospital stay. General infections (urinary tract infection, respiratory tract infection), flap anastomosis complications (venous or arterial), and wound complications (dehiscence, tissue necrosis, haematoma, chyle leak, salivary fistula or wound infection) were recorded. Infectious complications were judged using CDC criteria [19] and were considered significant if antibiotic therapy was instituted.

### 2.6. Statistical Analysis

The target sample size for this pilot study was 15 patients per group. Based on the CRP and IL-6 results of Braga *et al.* [20] in gastrointestinal cancer patients measured on POD1, this sample size yields a power >80% for detection of differences (at the 5% level) between the groups for these markers. 

Repeated measures analysis of variance (ANOVA) with asphericity correction was used to detect significant interaction between the effect of the treatment and the response over time for normally distributed variables. Inflammatory markers were log-transformed for statistical analysis. Postoperative length of hospital stay and intensive care unit stay were analyzed by logrank test and other between-group comparisons were assessed using the Mann-Whitney *U*-test or Student’s *t*-test as appropriate. *P* values <0.05 were considered to indicate statistical significance. Analyses were performed using SAS 8.2 (SAS Institute, Cary, NC). Data are presented as mean ± SEM or median (range).

## 3. Results

A total of eight patients were recruited (*n* = 4, IMN group; *n* = 4, STD group). Patient characteristics are detailed in Table 2. All patients underwent resection of a tumor located in the oral cavity with selective or radical neck dissection and required some form of flap reconstruction. Six patients required a tracheostomy tube (4 IMN, 2 STD) and all patients were tube fed postoperatively via an intra-operatively placed nasogastric tube. Prophylactic antibiotic treatment was given to all patients for 7 days postoperatively. The groups were well-balanced at baseline for mean body weight (STD: 66.6 ± 4.7 *vs.* IMN: 66.5 ± 4.2 kg). None of the patients was malnourished according to their total body protein status. The mean ratio of measured to pre-illness TBP was 0.97 ± 0.05 (range 0.83–1.30).

**Table 2:** Patient characteristics and clinical data.

Pt	Sex	Age	Staging	Diagnosis	Procedure		OP	LOS
					Excision	Reconstruction	*h*	*d*
*Standard group*								
A	F	79	Tx N1 Mx	Buccal melanoma	Wide local excision (WLE)	Forearm free flap	8	24
B	M	71	T2 N2	SCC floor of mouth	WLE, marginal mandibulectomy	Pectoralis pedicle flap	11	21
C	M	22	T2 N0 M0	SCC tongue	Hemiglossectomy	Forearm free flap	8	22
D	M	17	- ^a^	Ameloblastoma mandible	Segmental mandibulectomy	Fibular free flap	5.5	7
*Immunonutrition group*								
E	M	63	T2 N0 M0	SCC tongue	Hemiglossectomy	Forearm free flap	7	10
F	F	68	T4 N2	SCC floor of mouth	Segmental mandibulectomy	Fibular free flap	7.5	43
G	M	28	T2 N1	SCC tongue	Hemiglossectomy	Forearm free flap	8	10
H	M	46	- ^a^	ACC floor of mouth	WLE	Forearm free flap	8.5	9

OP = duration of operation; LOS = length of hospital stay; Pt = patient; WLE = wide local excision; SCC = squamous cell carcinoma; ACC = adenoid cystic carcinoma; ^a^ No staging system exists for the category of cancer.

Enteral feeding commenced a median 19.5 (range 9–20.5) h after the end of surgery in the IMN group and 22 (18.5–24) h in the STD group. The median daily energy intake postoperatively in the IMN group was 1590 (range 1542–1637) kcal for a median 6 (range 5–6) days. The corresponding figures for Isosource for the same time period in the STD group were 1404 (640–1570) kcal (*P* = 0.17). There was no difference in the average daily postoperative consumption of energy (*P* = 0.17) between the groups. Median daily protein intake in the IMN group (89.0, range 86–92 g) was significantly higher than in the STD group (50.3, 46–56 g; *P* = 0.0012).

**Figure 1:**
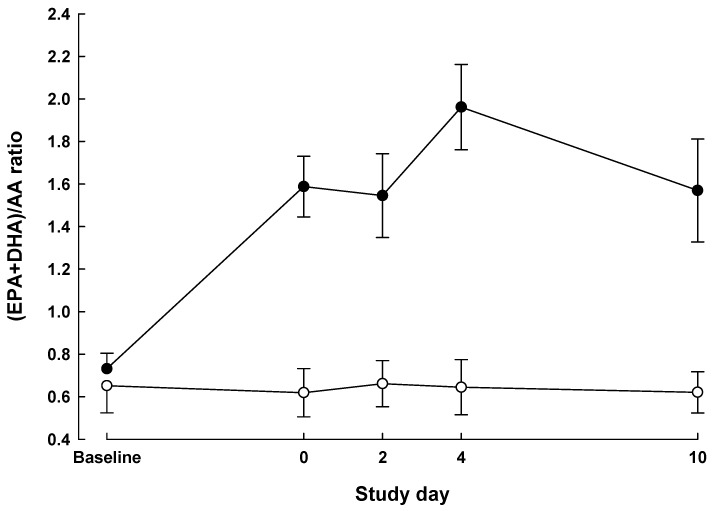
Plasma phosphatidylcholine (PC) (eicosapentaenoic acid (EPA) plus docosahexaenoic acid (DHA))/arachidonic acid (AA) ratio (mean ± SEM) measured at baseline, day of surgery and on postoperative days 2, 4 and 10 in patients who received Impact preoperatively and postoperatively (immunonutrition (IMN) *n* = 4, solid symbols) compared with patients who received no preoperative supplemental nutrition and Isosource postoperatively (standard treatment (STD) *n* = 4, open symbols).

The time profile for the (EPA + DHA)/arachidonic acid (AA) ratio in plasma PC differed significantly between the groups (*P* < 0.0001 for group × time interaction; Figure 1). This ratio did not differ between the groups at baseline (*P* = 0.62) but was significantly higher in the IMN group on day of surgery and at all postoperative time points (*P* < 0.01). As percent of total fatty acids, plasma PC EPA + DHA increased 2–3 fold in all IMN patients over the preoperative period.

**Figure 2:**
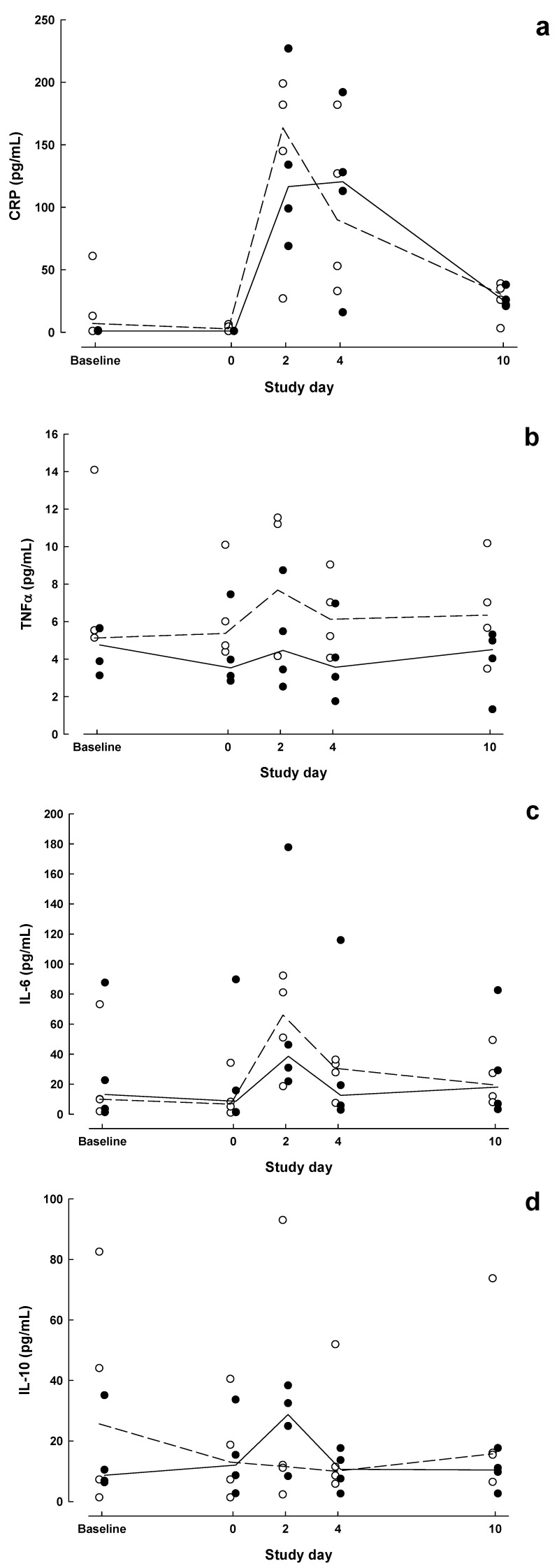
Inflammatory marker concentrations measured at baseline, day of surgery and on postoperative days 2, 4 and 10 in patients who received Impact preoperatively and postoperatively (IMN, solid symbols) compared with patients who received no preoperative supplemental nutrition and Isosource postoperatively (STD, open symbols). (**a**) C-reactive protein (CRP), (**b**) tumour necrosis factor (TNF)-α, (**c**) IL-6, (**d**) interleukin (IL)-10. Dashed (STD) and solid (IMN) lines connect median values.

Individual and median results for CRP, TNF-α, IL-6, and IL-10 are shown in Figure 2a–d. Group × time interactions were not significant for these markers (*P* > 0.13). CRP was significantly higher than baseline in both groups on POD2 (*P* < 0.05). 

IgG, IgA and IgM concentrations and total lymphocytes showed similar patterns of behavior with time in the two groups (Table 3). There was some evidence of a reduction from baseline for IgG and IgA on POD2 (*P* < 0.05) in the STD group which was not seen in the IMN group.

**Table 3:** Immunoglobulin G, A, M and total lymphocyte concentrations (mean ± SEM) at baseline, day of surgery (0) and on postoperative days 2, 4 and 10 in patients who received either Impact preoperatively and postoperatively (IMN) or Isosource postoperatively (STD).

	Baseline	Day 0	POD 2	POD 4	POD 10	*P* value ^a^		
						Group	Time	Group × Time
Immunoglobulin G (g/L)								
STD	11.03 ± 1.26	9.40 ± 1.19	6.93 ± 0.69 ^c^	7.36 ± 1.03	9.50 ± 1.47	0.37	0.0047	0.56
IMN	13.08 ± 2.68	12.28 ± 2.03	8.15 ± 0.61	8.83 ± 0.91	10.25 ± 1.32			
Immunoglobulin A (g/L)								
STD	3.33 ± 0.09	2.88 ± 0.17	2.10 ± 0.14 ^b^	2.43 ± 0.26	3.43 ± 0.21	0.15	0.0001	0.35
IMN	2.85 ± 0.10	2.70 ± 0.11	1.93 ± 0.26	2.35 ± 0.28	2.80 ± 0.16			
Immunoglobulin M (g/L)								
STD	1.34 ± 0.49	1.32 ± 0.43	0.80 ± 0.26	0.86 ± 0.27	1.67 ± 0.32	0.36	0.0028	0.17
IMN	0.90 ± 0.27	0.88 ± 0.31	0.57 ± 0.21	0.65 ± 0.22	0.89 ± 0.25			
Total lymphocytes (10^9^/L)								
STD	2.05 ± 0.56	2.05 ± 0.11	1.12 ± 0.30	1.23 ± 0.19	1.66 ± 0.34	0.98	0.026	0.56
IMN	1.89 ± 0.16	1.63 ± 0.25	1.43 ± 0.28	1.43 ± 0.10	1.80 ± 0.35			

Data are expressed as mean ± SEM; ^a^ Two-way repeated measures analysis of variance; ^b^ *P* < 0.05 for paired *t*-test *vs.* the preceding measurement; ^c^ *P* < 0.01 for paired *t*-test *vs.* the preceding measurement.

The median time spent in the intensive care unit was 18.5 (range 18–20) h in the IMN group and 19.5 (18–24) h in the STD group (*P* = 0.33). Median length of hospital stay was 10 (range 10–43) day in the IMN group and 21.5 (7–24) day in the STD group (*P* = 0.90; Table 1). Five postoperative complications developed in three patients in the STD group and two (wound infections) in one patient in the IMN group. Two patients developed infectious complications in the STD group. 

## 4. Discussion

This pilot study was not large enough to generate definitive results for inflammatory and immune markers or for clinical outcome. It provides indications only that an immune-modulating feed administered perioperatively to well-nourished patients undergoing surgery for head and neck cancer may suppress circulating pro-inflammatory cytokine concentrations along with CRP while enhancing the concentration of IL-10, an anti-inflammatory mediator, in the immediate postoperative period. In addition, over this early period (POD2), trends for less suppression of immunoglobulin production and total lymphocyte numbers were seen in the immunonutrition group. The overall pattern of results including clinical outcomes is supportive of benefit from perioperative treatment with fish oil and arginine enriched nutrition. A larger trial is needed, of course, to confirm this. Notably, every patient in the immunonutrition group showed increased plasma *n*-3 fatty acid concentrations on day of surgery, consistent with ingestion of the oral supplement, and these levels were elevated well above those in the standard feed group out to POD10, well beyond the 5–6 days of postoperative intake of the immunonutrition formula. 

Our target sample size of 30 patients was not reached due to a much lower recruitment rate than the projected four patients per month. This was due to several factors: The quite restrictive patient inclusion criteria, which required normal nutritional status and no previous wide-field radiotherapy, reduced the eligibility rate to around two patients per month. A further halving of this rate occurred because of the logistical difficulties of arranging the baseline body protein scans on patients outside the local area. Finally, a prolonged ethics and institutional approval process limited the time allocated for the work to approximately 8 months because of budgetary constraints.

A number of randomized studies have investigated the effects of single immune-modulating nutrients on post-surgery inflammatory and immune markers and clinical outcome in head and neck cancer patients. Post-surgery enteral nutrition enriched with arginine has been used in most studies [21,22,23,24,25,26] on the basis of its role in wound healing and immune function [27,28] and has been shown to confer beneficial immune effects when provided postoperatively to patients undergoing major surgery for cancer [29]. In head and neck surgery patients the potential beneficial effects of arginine administration on inflammatory and immune markers assessed from POD5 and beyond was not generally evident [21,22,23,24] except in one study [26] that showed improved lymphocyte counts and CD4/CD8 ratios on PODs 4 and 8 in the arginine group compared to control patients. Reduced wound complications [21,23] and postoperative length of stay [23] were reported with arginine administration. Riso *et al.* [26] reported improved clinical outcome in terms of reduced postoperative complications and length of hospital stay in a malnourished subgroup of patients. Arginine content of the feed in these head and neck surgery studies was 6.25 g/L, half the concentration used in our study, except for the higher dose trial (8.5 g/L) of de Luis *et al.* [25]. 

One study only has examined perioperative treatment of head and neck surgical patients with arginine-enriched feeds and no significant effect on clinical outcome was reported [30]. No study has reported use of *n*-3 fatty acid enriched nutrition provided as a single immunonutrient in this category of patients, except after discharge from hospital [31]. Nutritional supplements enriched in a combination of *n*-3 fatty acids from fish oil and arginine have been shown to be particularly beneficial in gastrointestinal surgical patients [1,2,3,4]. We are aware of only three published randomized trials that examined this combination of immunonutrients in head and neck surgery patients [12,13,15]. All used Impact but clinical outcome was demonstrably improved only when the formula was provided perioperatively [12,15]. None of these studies provided evidence of raised serum or biomembrane *n*-3 fatty acid levels in the early postoperative period in the immunonutrition groups. All the head and neck surgery studies mentioned, with two exceptions where malnourished patients were studied [24,30], recruited patients who were not selected according to nutritional status. While immunonutrition may be especially beneficial to malnourished patients undergoing surgery, since malnutrition is associated with immune suppression [32], it is expected also to benefit the well-nourished in view of the immune suppression and inflammation that follow a major surgical insult. In our otolaryngology center, patients assessed as being malnourished are routinely provided with preoperative immunonutrition (Impact) and we hypothesized in designing the present study that such treatment may show beneficial effects in the non-malnourished patients.

An obvious weakness of the current study is the small sample size and consequent lack of power in establishing treatment efficacy for any of the endpoints. Strengths of the study include: (1) an objective determination of malnutrition by direct measurement of body protein stores, an approach we have applied in other patients with chronic disease [33]; (2) determination of plasma levels of EPA and DHA which provide evidence for likely incorporation of these fatty acids into cell membranes [34] where they exert their biological effects [35]; (3) both pre- and postoperative immunonutritional treatment. In addition we assessed inflammatory and immune markers in the very early postoperative period (POD2) rather than 8 day after surgery as was the case in the earlier perioperative Impact study [15]. However, peak IL-6 concentrations would most probably be observed before POD2 given the short half-life of this cytokine and the fact that it induces the hepatic synthesis of CRP [36]. 

## 5. Conclusions

This is the first study to investigate the potential beneficial effects of a fish oil and arginine enriched immunonutritional formula in well-nourished patients undergoing surgery for head and neck cancer. The size of the study precluded definitive conclusions regarding these effects on post-surgery inflammation and immune function. However, the pattern of changes seen as a result of the treatment was consistent with improved inflammatory and immune status. These changes may be expected to underlie clinically beneficial outcomes and the results of this study for postoperative length of hospital stay and complications support that contention.
